# Leveraging practice-based research networks to accelerate implementation and diffusion of chronic kidney disease guidelines in primary care practices: a prospective cohort study

**DOI:** 10.1186/s13012-014-0169-x

**Published:** 2014-11-23

**Authors:** James W Mold, Cheryl B Aspy, Paul D Smith, Therese Zink, Lyndee Knox, Paula Darby Lipman, Margot Krauss, D Robert Harris, Chester Fox, Leif I Solberg, Rachel Cohen

**Affiliations:** Department of Family and Preventive Medicine, University of Oklahoma Health Sciences Center, 900 NE 10th Street, Oklahoma City, OK 73104 USA; University of Wisconsin School of Medicine and Public Health, 1100 Delaplaine Court, Madison, WI 53715 USA; Boonshoft School of Medicine, Wright State University, 3640 Colonel Glenn Highway, Dayton, OH 45435 USA; Los Angeles Practice-Based Research Network (LA Net), 3940-B East Broadway, Long Beach, CA 90803 USA; Westat, 1600 Research Boulevard, Rockville, MD 20850 USA; State University of New York at Buffalo, 1315 Jefferson Avenue, Buffalo, NY 14208 USA; HealthPartners Institute for Education and Research, Mail Stop 23301, P.O. Box 1524, Minneapolis, MN 55440-1524 USA

**Keywords:** Implementation, Diffusion, Primary care, Practice-based research network, Chronic kidney disease

## Abstract

**Background:**

Four practice-based research networks (PBRNs) participated in a study to determine whether networks could increase dissemination, implementation, and diffusion of evidence-based treatment guidelines for chronic kidney disease by leveraging early adopter practices.

**Methods:**

Motivated practices from four PBRNs received baseline and periodic performance feedback, academic detailing, and weekly practice facilitation for 6 months during wave I of the study. Each wave I practice then recruited two additional practices (wave II), which received performance feedback and academic detailing and participated in monthly local learning collaboratives led by the wave I clinicians. They received only monthly practice facilitation. The primary outcomes were adherence to primary care-relevant process-of-care recommendations from the National Kidney Foundation Kidney Disease Outcomes Quality Initiative Guidelines. Performance was determined retrospectively by medical records abstraction. Practice priority, change capacity, and care process content were measured before and after the interventions.

**Results:**

Following the intervention, wave I practices increased the use of ACEIs/ARBs, discontinuation of NSAIDs, testing for anemia, and testing and/or treatment for vitamin D deficiency. Most were able to recruit two additional practices for wave II, and wave II practices also increased their use of ACEIs/ARBs and testing and/or treatment of vitamin D deficiency.

**Conclusions:**

With some assistance, early adopter practices can facilitate the diffusion of evidence-based approaches to other practices. PBRNs are well-positioned to replicate this process for other evidence-based innovations.

**Electronic supplementary material:**

The online version of this article (doi:10.1186/s13012-014-0169-x) contains supplementary material, which is available to authorized users.

## Background

Many practice-based research networks (PBRNs) function as learning communities that strive to improve quality of care in member practices [1-4]. Some provide resources to support dissemination and implementation, such as performance feedback, identification and spread of best indigenous practices, academic detailing, and practice facilitation, evidence-based strategies that have been tested and refined in Ontario, Canada, the Oklahoma Physicians Resource/Research Network (OKPRN), and in other primary care settings [5-11]. Respectful relationships between the academic detailers and practice facilitators (PFs), and the practice clinicians and staff, appear to be critical to success. This principle has also been the key to the success of Cooperative Extension, in which extension agents develop relationships with farmers and their families to facilitate implementation of innovative farming practices [12].

While this approach to dissemination and implementation has been successful in practices that volunteer to participate, questions remain about how to engage other practices: whether the rate of diffusion can be increased and whether the cost of implementation assistance can be reduced. Evidence that peer-to-peer learning can motivate practice improvement, especially when enhanced by competition, has led to the development and use of learning collaboratives [13-15]. These collaboratives typically involve large numbers of practices and include periodic conferences between periods of local improvement activities. In OKPRN, researchers working with small rural practices have adapted this approach, holding shorter more frequent meetings with a smaller number of practices [16]. These local learning collaboratives (LLCs) are less costly and probably better-accepted by clinicians and staff than the larger, more formal collaboratives [17].

The Agency for Healthcare Quality and Research wanted to determine whether PBRNs could speed dissemination and implementation of evidence-based practices. We were one of the several groups to receive funding for this initiative. We chose to address chronic kidney disease (CKD) guidelines, since member practices indicated that this was an area of both weakness and interest. Our goal was to try to help a group of early-adopter practices implement the guidelines using standard implementation strategies, then see whether those practices could recruit and train additional practices using LLCs and less intensive facilitation.

Between the 1988–1994 and the 2003–2006 National Health and Nutrition Examination Surveys, the prevalence of CKD in individuals aged 60 and older increased from 18.8% to 24.5% [18]. CKD is associated with a 5-year all-cause mortality rate of 24% and a 20% 5-year requirement for transplant or dialysis [19]. Evidence-based guidelines for the management of patients with CKD have been available to practicing physicians since 2002 [20], and there is some evidence to suggest that guideline-based care can delay CKD progression and reduce mortality [13,21-24]. However, dissemination, implementation, and diffusion of guideline recommendations have been suboptimal [16,25,26].

The primary aim of the present study was to determine the extent to which ‘early adopter’ practices could facilitate recruitment of other practices and, using the experience gained during their own implementation efforts, help the new practices to implement guideline recommendations. Success was measured by numbers of wave II practices enrolled; numbers of LLCs formed, held, and attended by wave I practice representatives; and improvements in performance by wave II practices. In other words, we wanted to determine if we could speed up the diffusion of ‘best practices’ by leveraging the relationships and resources of practice-based research networks.

The secondary aim was to determine whether the change processes used by wave II practices would be the same as or different from those used by the early adopters. The outcome measures for this aim were changes in priority for improvement of CKD care, change process capability, and care process content, concepts proposed by Solberg [27].

## Methods

### Study design

This was a cohort study involving primary care practices enrolled in two waves. Data on practice enrollment, practice characteristics, and practice change component scores were captured concurrently. Practice performance data were collected retrospectively after each wave using medical records abstraction. Additional information was provided by concurrent facilitator field notes and retrospective clinician interviews. Four PBRNs participated in the project: OKPRN, the Los Angeles Practice-Based Research Network (LA Net), the Minnesota Academy of Family Physicians Research Network (MAFPRN), and the Wisconsin Research and Education Network (WREN). A coordinating center, Westat, managed the project activities and conducted the data management and analysis.

Each PBRN was asked to recruit eight member practices (*N* = 32) to participate in wave I of the study. Since each network handled this differently, we have no data on the number of practices who declined to participate. At the time of enrollment, a clinician from each of these practices agreed to help the investigators recruit two additional practices (*N* = 64) and help to facilitate the LLC strategy during wave II. Each PBRN provided a local project coordinator, an academic detailer, and PFs (1 full-time equivalent) to work directly with the practices, collect performance data, and, during wave II, organize the monthly LLC meetings. In addition to practice performance data, surveys completed by a lead clinician from each practice were used to collect information about the impact of the implementation and diffusion strategies on three components of practice change: priority for change, change process capability, and care process content [28].

Practices were directed to focus on the eight processes of care emphasized in the National Kidney Foundation Kidney Disease Outcomes Quality Initiative Guidelines: 1) making and documenting the diagnosis, 2) ordering appropriate tests based upon severity of disease, 3) discontinuing potentially harmful medications, 4) starting potentially beneficial medications, 5) managing diabetes and cardiovascular disease risk factors, 6) educating patients about vein preservation, 7) giving appropriate immunizations, and 8) referring patients with advanced disease to a nephrologist [20]. Specific recommendations within each of these categories were consolidated into a one-page, laminated decision-support tool, which was provided to the practices and discussed in the academic detailing sessions.

Maintenance of Certification (MOC) Part IV credit was provided through the American Boards of Family Medicine and Internal Medicine, which was a significant motivator for clinician participation. Continuing Medical Education (CME) credit was also provided through the American Academy of Family Physicians.

The project was approved by the Institutional Review Boards at the University of Oklahoma Health Sciences Center, the University of Wisconsin at Madison, the University of Minnesota, the University of Southern California Health Sciences Center, and Westat.

### Interventions

#### Wave I

At the start of wave I, practices received performance evaluation and feedback reports and academic detailing, followed by *weekly* practice facilitation for 6 months. Academic detailing included a review of the guideline recommendations, a discussion of the one-page decision-support tool (included as an attachment), a forum to discuss questions and concerns, a review of the practice’s current methods and performance, and guidance from the methods used in high performing practices, culminating in an agenda for improvement. The PFs were expected to spend 2–4 h per *week* in each practice helping clinicians and staff implement the agreed-upon changes in their care processes, teaching them how to do quality improvement (QI) (e.g., plan-do-study-act cycles, QI teams, staff meetings, etc.), and collecting ‘unofficial’ baseline and monthly performance data for their use. The PBRNs worked with these practices to recruit and enroll two additional practices in close geographic proximity to the wave I practices, and typically belonging to similar health systems or using an electronic health record (EHR) from the same vendor.

#### Wave II

Practices in wave II also received performance evaluation and feedback and academic detailing. The three practices in each cluster (one wave I, two wave II) were expected to participate in a 1-h LLC meeting each month to review each other’s performance data, discuss lessons learned, and share EHR templates, order sets, and other tools and materials they had discovered or created (e.g., patient education handouts). The basic agendas for the LLC sessions were preset, including review of interim performance data in each participating practice and discussion of successful and unsuccessful approaches tried and obstacles and opportunities encountered. The first one or two LLCs often focused on the methods adopted by the wave I practices, with arrangements for sharing checklists, templates, and order sets. The PFs generally handled administrative aspects of the LLCs, and the wave I clinicians tended to lead and facilitate the discussions.

During this wave, the PFs spent 2–4 h per month helping wave II practices implement desired changes in their care processes, teaching them about QI, and abstracting performance data for QI purposes. Our hope was that the assistance provided by wave I practices during the LLC meetings would reduce the need for PF visits and that monthly, rather than weekly, visit would be sufficient. This was based only upon our collective judgment, not on specific data.

### Training and standardization

Training was provided for the academic detailers and PFs prior to the interventions. The academic detailer training focused on the guideline recommendations and suggestions regarding implementation. The PF training addressed QI concepts, practice facilitation and group facilitation skills, chart auditing and feedback, the Chronic Care Model, the nature and behavior of complex adaptive systems, MOC and CME logistics, and the CKD guidelines (including the toolkit and implementation suggestions) and project-related data collection and data transfer. Prior to wave II, PFs received additional training on the facilitation of LLCs and were provided a sample LLC agenda to further standardize the experience for the participating clinicians. PFs also participated in training on collection of the retrospective medical record data upon which these primary analyses are based.

### Practice performance measurement

After completion of each wave, the PFs screened medical records of diabetic patients between 50 and 84 years of age to identify at least 30 patients with two estimated glomerular filtration rates (eGFR) <60, at least 3 months apart for the next phase of abstraction. If 30 patients meeting these criteria could not be identified, PFs were instructed to search the records of patients between 50 and 84 years of age with a diagnosis of hypertension. If 30 patients still could not be identified, PFs were instructed to identify patients already diagnosed with CKD. The PFs then abstracted the medical records of patients who had evidence of CKD to collect information relevant to practice performance during the specified time periods of interest. Data on the following were abstracted for every visit during the qualifying time period: 1) CKD diagnosis defined as an eGFR staying below 60 for more than 3 months; 2) A1c measurement at least annually, 3) LDL cholesterol measurement at least annually, 4) microalbumin measurement at least annually, 5) Hgb measurement annually if eGFR <45, 6) ACEI or ARB initiation, 7) discontinuation of NSAIDS, 8) vitamin D measurement and/or initiation of vitamin D supplements if eGFR <45, and 9) referral to a nephrologist for any eGFR <30. We also examined changes in LDL, A1c, and the mean of the last three diastolic (DBP) and systolic blood pressures (SBP) before and after the intervention.

The abstraction windows for wave I practices were the 12 months prior to the beginning of the intervention (pre-intervention) and the 12 months after the *start* of the intervention (post-intervention). The abstraction windows for wave II practices were the 12 months prior to the beginning of the intervention (pre-intervention) and the *9 months* after the *beginning* of the intervention (post-intervention).

### Practice contexts and measures of change in priorities and processes

Data on organizational characteristics (context) were collected from practices upon enrollment. *Priority for improving CKD care* was measured on a visual analog scale (*CKD priority scale*). The instructions read, ‘Considering all the priorities your clinic has over the next year (e.g., EHR, financial goals, QI of various conditions, physician recruitment), what is the priority for your clinic to improve CKD care (on a scale of 0–10, where 0 = not a priority, 5 = medium priority, and 10 = highest priority of all)?’

*The Change Process Capability Questionnaire (CPCQ)*, developed and tested by Solberg [27], was used to measure organizational factors associated with readiness of the practice to manage changes needed to implement guideline recommendations. The instrument includes 30 items measured on a five-point Likert scale, from strongly agree to strongly disagree. Three domain scores under organizational factors include ‘previous history of change’, ‘plans for organizational refinement’, and ‘ability to initiate and sustain change’. The strategies (‘strategy count’) are specific approaches to managing change, such as periodic measurement of performance or delegating physician work to non-physician staff.

*The Physician Practice Connections—Patient-Centered Medical Home Research Version (PPC-PCMH-R)*, developed by the National Committee for Quality Assurance, assessed the implementation of key practice systems for chronic illness management, including health systems (e.g., QI processes), information systems (e.g., registries), decision support (e.g., structured visits), delivery system design (e.g., teamwork), and patient self-management support.

Survey data were collected at the beginning and end of each wave including the priority for improving CKD care and CPCQ data from the lead clinician, and one PPC-PCMH-R from each practice. The CPCQ measures readiness to change and use of various change process strategies, while the PPC-PCMH-R measures actual practice systems put into place to achieve better performance through the use of that readiness and those strategies.

### Analytic methods

Except as noted below, all analyses were preplanned. Descriptive statistics (frequencies and proportions) were used to describe the wave I and wave II practice characteristics; comparisons between waves were made using Fisher’s exact test. The proportion of patients with CKD on the problem list in the pre-intervention period was compared to the proportion with CKD on the problem list post-intervention but not pre-intervention. Confidence intervals (CI) were calculated. The proportions of patients with eGFR <30 who were referred to a nephrologist before and after the interventions were similarly compared. The McNemar test was used to assess changes in guideline implementation with respect to prescribing ACEI/ARB, discontinuing NSAIDS and A1c, Hgb, and vitamin D testing (or supplementation), while the paired *t* test was used to assess changes in A1c, SBP and DBP, and LDL test results.

To account for possible correlation of repeated measures within patients, a generalized estimating equation (GEE) approach was used to fit repeated measures models to assess the pre- to post-intervention change in the implementation of the guideline recommendations. Except for Hgb and vitamin D measurements, the modeling analyses were restricted to patients with evidence of CKD prior to the intervention (at least 3 months of eGFR <60 or documentation of CKD in the problem list). For Hgb and vitamin D measures, the modeling was further restricted to those subjects with an eGFR <45 in both periods. Only those patients with outcome information available during both the pre- and post-intervention periods contributed to the models. Changes in implementation of the CKD guidelines were modeled separately for wave I and wave II practices.

Since preliminary analyses indicated that CKD recognition (CKD reported in the problem list in the medical record) might have influenced the implementation of the other guideline recommendations, the models were fit to the data for each study outcome to assess the effect of the intervention, adjusted for any influence of when CKD was recognized and the interaction of the intervention with CKD recognition, if any (Tables 1 and 2). A significant main effect of intervention would indicate that implementation of the guideline changed significantly over time, independent of CKD recognition or the timing of CKD recognition. Assuming the change to be in the desired direction, the result would suggest that the intervention was effective in helping the practices to implement the guideline. No other variables were included in the models for this feasibility study.

**Table 1:** **Wave I modeling of intervention effect, adjusted for timing of recognition of CKD diagnosis in the medical record**

**Model effects**	**CKD guideline**						
	**HbA1c measured**	**ACEI/ARB prescribed**	**Taking NSAIDS**	**Hgb measured**		**Vitamin D measured**	**Alb/Cr ratio measured**
	**OR (95% CI)**	**OR (95% CI)**	**OR (95% CI)**	**OR (95% CI)**	**OR (95% CI)**	**OR (95% CI)**	**OR (95% CI)**
Intervention effect (post- vs. pre-intervention)	NA	1.3 (1.1–1.6)	0.7 (0.4–1.0)	1.7 (1.1–2.6)	0.9 (0.7–1.3)	2.6 (1.7–3.8)	NA
*p* value		0.001	0.030	0.014	0.68	<0.0001	
CKD recognition:	NA						NA
Recognized pre-intervention vs. not recognized		0.9 (0.5–1.6)	0.9 (0.3–2.6)	2.9 (0.9–9.5)	1.1 (0.6–1.9)	0.9 (0.2–3.4)	
Recognized post-intervention vs. not recognized		0.8 (0.4–1.6)	0.8 (0.2–2.6)	2.5 (0.7–8.6)	1.6 (0.9–3.1)	1.0 (0.3–3.9)	
Recognized pre-intervention vs. recognized post-intervention		1.1 (0.7–1.7)	1.2 (0.5–2.7)	1.2 (0.6–2.2)	0.7 (0.4–1.1)	0.9 (0.5–1.7)	
*p* value		0.84	0.92	0.30	0.18	0.95	
Interaction of intervention with CKD recognition:							
Post- vs. pre-intervention for CKD recognized pre-intervention	1.4 (1.0–1.9)						1.6 (1.1–2.1)
Post- vs. pre-intervention for CKD recognized post-intervention	1.1 (0.8–1.6)						1.2 (0.7–1.9)
Post- vs. pre-intervention for CKD not recognized	0.6 (0.3–1.0)						0.5 (0.3–0.9)
*p* value	0.037						0.004

*NA* not applicable (The main effects contributing to an interaction have different interpretations in the presence of the interaction).

**Table 2:** **Wave II modeling of intervention effect adjusted for timing of recognition of CKD diagnosis in the medical record**

**Model effects**	**CKD guideline**			
	**HbA1c measured**	**ACEI/ARB prescribed**	**Taking NSAIDS**	**Vitamin D measured**
	**OR (95% CI)**	**OR (95% CI)**	**OR (95% CI)**	**OR (95% CI)**
Intervention effect (post- vs. pre-intervention)	1.0 (0.8–1.2)	1.4 (1.2–1.6)	0.9 (0.7–1.2)	2.2 (1.7–2.9)
*p* value	0.84	<0.001	0.35	<0.0001
CKD recognition: recognized pre-intervention vs. not recognized	1.4 (0.9–2.2)	1.2 (0.8–1.8)	0.8 (0.4–1.6)	2.3 (0.8–7.0)
Recognized post-intervention vs. not recognized	1.5 (0.9–2.5)	1.3 (0.8–2.0)	1.0 (0.5–2.3)	2.5 (0.8–7.9)
Recognized pre-intervention vs. recognized post-intervention	0.9 (0.6–1.4)	1.0 (0.7–1.3)	0.8 (0.4–1.4)	0.9 (0.6–1.5)
*p* value	0.24	0.51	0.67	0.30

The initial modeling of the wave II data included all study practices. However, there were a number of wave II practices that attended none or very few of the LLC sessions. These practices would not be expected to benefit from the diffusion strategy, possibly leading to underestimation of the effect of the intervention when included. Consequently, a sub-analysis was conducted on those 30 practices that participated in at least five LLC sessions to assess the stability of the model estimates (sensitivity analysis).

Differences between measures of priority and change capacity and care processes between baseline and post-intervention were estimated using the paired *t* test. All analyses were carried out using SAS software system, version 9.2 (SAS Institute Inc., Cary, NC, USA). Imputation for missing values was not considered nor was there any correction made in the analyses for multiple comparisons.

## Results

### Practice participation and characteristics

Across the four PBRNs, 32 practices were recruited for wave I. One practice had to delay entry into the project and was moved to wave II, leaving 31 practices that participated in wave I. All wave I practices had been members of one of the four PBRNs prior to this project.

Following the wave I implementation intervention, the 31 wave I practices were able to help the research team recruit and enroll a total of 58 wave II practices (mean: 1.9 per practice). Wave II practices were statistically less likely to be a member of a PBRN and to have participated in a QI project before, but they were otherwise similar to wave I practices with regard to the characteristics examined. A comparison of wave I and wave II practices, with characteristics categorized as suggested by Damshroeder et al., can be found in Table 3 [29].

**Table 3:** **Practice characteristics by study wave**

**Characteristic**	**Wave I**	**Wave II**	***p*** **value** ^**c**^
	**(** ***N*** **= 31** ^**a**^ **)**	**(** ***N*** **= 58** ^**b**^ **)**	
Outer setting			
Practice type: *n* (%)			
Academic practice	3 (10)	5 (10)	0.4
Federally designated health center or rural clinic	9 (30)	20 (39)	
Hospital outpatient practice	5 (17)	6 (12)	
Private practice	8 (27)	19 (37)	
Other (Free Clinics, etc.)	5 (16)	2 (4)	
Missing	1	6	
Practice location: *n* (%)			
Rural	11 (38)	15 (29)	0.7
Suburban	6 (21)	14 (27)	
Urban	12 (41)	23 (44)	
Missing	2	6	
Clinician owned: *n* (%)			
Yes	8 (27)	13 (25)	1.0
No	22 (73)	40 (75)	
Missing	1	5	
PBRN member			
Yes	31 (100)	39 (67)	0.0002
No	0	19 (33)	
Inner setting			
Medical record type: *n* (%)			
EHR	28 (93)	43 (81)	0.2
Paper	2 (7)	10 (19)	
Missing	1	5	
Number of full-time clinicians: *n* (%)			
1	4 (14)	8 (16)	0.7
2	5 (18)	7 (14)	
3–5	10 (36)	12 (24)	
≥6	9 (32)	22 (45)	
Missing	3	9	
Mid-level practitioners: *n* (%)			
Yes	19 (63)	35 (66)	0.8
No	11 (37)	18 (34)	
Missing	1	5	
Ever in QI project: *n* (%)			
Yes	25 (89)	31 (65)	0.0292
No	3 (11)	17 (35)	
Missing	3	10	

^a^One wave I practice did not provide any baseline information.

^b^One wave II practice did not provide any baseline information.

^c^ *p* values obtained from Fisher’s exact test.

*EHR* electronic health record, *eGFR* estimated glomerular filtration rate, *QI* quality improvement, *PF* practice facilitator, *LLC* local learning collaborative.

Twenty of the expected 30 LLCs actually met as planned. The predominantly inner-city safety net practices in Los Angeles found it impossible to schedule LLCs and instead held monthly conference calls to which all participating practices were invited. In OKPRN, MAFPRN, and WREN, eight, seven, and seven LLCs were formed and met a total of 121 times. Wave I clinicians attended 82% of the LLC meetings.

### Wave I practice performance

Wave I practice performance data were obtained from the medical records of 711 patients. The majority were female (62%), born prior to 1/1/45 (54%) and thus were Medicare eligible, and white (78%). Among the 271 patients with evidence of CKD during the pre-intervention period, 39% (95% CI: 33%–45%) had CKD in the problem list compared to 66% (95% CI: 62%–70%) of 461 meeting CKD criteria during the post-period.

Among the 105 patients with eGFR <30 during the pre-intervention period, 66% (95% CI: 56%–75%) had a referral to a nephrologist, compared to 71% (95% CI: 62%–79%) of 124 with eGFR <30 during the post-intervention period. In the unadjusted analysis, prescription of ACE/ARBs and testing for Hgb and vitamin D (or supplementation) increased significantly from the pre- to post-intervention period (Table 4).

**Table 4:** **CKD performance outcome measures for wave I and wave II practices**

**Performance measure**	**Wave I**			**Wave II**		
	**Pre-intervention period**	**Post-intervention period**	***p*** value*	**Pre-intervention period**	**Post-intervention period**	***p*** value*
If eGFR <60	*N* = 374			*N* = 660		
ACE or ARB prescribed: *n* (%)	232 (63%)	255 (69%)	0.002	354 (55%)	407 (63%)	<0.001
Taking NSAIDs: *n* (%)	25 (7%)	18 (5%)	0.10	51 (8%)	45 (7%)	0.44
A1c testing performed: *n* (%)	288 (77%)	297 (79%)	0.25	492 (89%)	487 (88%)	0.65
Urine microalbumin test performed: *n* (%)	158 (42%)	175 (47%)	0.16	NA	NA	NA
LDL test performed: *n* (%)	309 (83%)	305 (82%)	0.75	NA	NA	NA
A1c test results: Mean (SD)	7.1% (1.4)	7.2% (1.5)	0.78	7.4% (1.6)	7.3% (1.5)	0.07
Systolic BP measure (mmHg): mean (SD)	132.5 (16.3)	132.8 (16.0)	0.72	132.0 (15.9)	131.4 (14.9)	0.39
Diastolic BP measure (mmHg): mean (SD)	72.8 (9.1)	72.7 (9.0)	0.93	72.0 (9.4)	71.7 (8.5)	0.48
LDL test result (mg/dl): mean (SD)	89.8 (36.6)	90.2 (32.8)	0.84	96.3 (38.1)	92.0 (34.5)	0.004
If eGFR <45	*N* = 138			*N* = 233		
Hemoglobin test performed: *n* (%)	84 (61%)	100 (72%)	0.02	NA	NA	NA
Vitamin D testing (or supplement): *n* (%)	51 (37%)	83 (60%)	<0.001	87 (37%)	132 (57%)	<0.001

*SD* standard deviation, *NA* not applicable because it was an annual recommendation and in wave II, the post-intervention period was only 9 months.

*McNemar’s test was used to generate *p* values for categorical-scaled outcomes and the paired *t* test for continuous-scaled outcome measures.

The interaction of the intervention with CKD recognition contributed significantly to predicting the probability of A1c measurement in the adjusted modeling (*p* = 0.037), indicating that the effect of the intervention varied according to whether or not CKD was recognized in the patient and the timing of such (Table 1). Among participants with CKD recognized in the pre-intervention period, there was a significantly increased probability of having HbA1c measured in the post-intervention period compared to the pre-intervention period (odds ratio (OR) = 1.4, 95% CI: 1.0–1.9). This was also true for the measurement of microalbumin, with the interaction contributing significantly to the model (*p* = 0.004); participants with CKD recognized in the pre-intervention period were significantly more likely to have microalbumin measured in the post-intervention period compared to the pre-intervention period (OR = 1.6, 95% CI: 1.1–2.1).

The modeling results indicate that the probability of being prescribed ACEIs/ARBs and having Hgb and vitamin D measured (or vitamin D supplements given) increased significantly from pre- to post-intervention (OR = 1.3, 95% CI: 1.1–1.6, *p* = 0.001; OR = 1.7, 95% CI: 1.1–2.6, *p* = 0.014; and OR = 2.6, 95% CI: 1.7–3.9, *p* <0.0001, respectively), but that the probabilities did not differ according to whether or not CKD was recognized or the timing of such (*p* ≥0.30). The odds of being on NSAIDS was significantly lower in the post-intervention period compared to the pre-intervention period (OR = 0.7, 95% CI: 0.4–0.95; *p* = 0.030) but did not differ according to whether or not CKD was recognized or the timing of such (*p* = 0.92).

### Wave II practice performance

For Wave II, data were abstracted on 1,179 patients. The majority were female (63%), Medicare eligible (60%) and white (78%), similar to wave I patients. Among the 466 patients with evidence of CKD during the pre-intervention period, 41% (95% CI: 37%–45%) had CKD in the problem list compared to 58% (95% CI: 54%–62%) of 664 with CKD evidence during the post-intervention period.

Among the 129 patients with any eGFR <30 during the pre-intervention period in wave II, 60% (95% CI: 51%–68%) had a referral to a nephrologist, compared to 55% (95% CI: 47%–63%) of 176 patients with eGFR <30 first documented during the post-intervention period. The prescription of ACEI/ARBs and testing for vitamin D (or supplementation) among those with eGFRs <45 increased significantly post-intervention (Table 4).

In the adjusted modeling, the probability of having Hgb measured did not differ significantly from zero (no change pre- to post-intervention) (OR = 0.8, 95% CI: 0.6–1.2, *p* = 0.38), but did differ in relation to CKD recognition (*p* = 0.009) (Table 2). Patients with CKD recognized in the pre-intervention period were significantly less likely to have Hgb measured in the post-intervention period compared to pre-intervention period than those with CKD recognized in the post-intervention period (OR = 0.4, 95% CI: 0.2–0.8; *p* = 0.006).

The probability of being prescribed ACEIs/ARBs increased significantly from pre- to post-intervention (OR = 1.4, 95% CI: 1.2–1.6; *p* <0.0001), as did measurement of vitamin D (or vitamin D supplements given) (OR = 2.2, 95% CI: 1.7–2.9; *p* <0.0001) (Table 2). The probability of implementation of these guidelines did not differ according to whether or not CKD was recognized or the timing of such (*p* ≥0.3). The probability of having microalbumin measured increased from the pre- to post-intervention period, but the change was of only borderline significance (OR = 1.2, 95% CI: 1.0–1.5; *p* = 0.069). CKD recognition did not contribute to predicting the change in microalbumin measurement (*p* = 0.36).

Sub-analyses were conducted on those 30 practices that attended at least five LLC sessions; ten practices attended five (all included wave I staff members) and 20 practices attended six (12 wave I practice staff members attended six; eight attended five). These analyses included 387 subjects with information available during the pre- and post-intervention periods; this number was further reduced to 125 subjects for the Hgb and vitamin D modeling which required eGFR to be <45. As was true for the original wave II modeling, the prescribing of ACEIs/ARBs increased significantly for the post-intervention period compared to pre-intervention period for this subset of practices (OR = 1.3, 95% CI: 1.1–1.5, *p* = 0.005), as did measurement of vitamin D (OR = 2.4, 95% CI: 1.7–3.4, *p* <0.0001). Measurement of A1c (*p* = 0.81), Hgb (*p* = 0.84), and microalbumin (*p* = 0.67) did not differ significantly according to the study period in either modeling or did the use of NSAIDS (*p* = 0.68).

### Changes in eGFR

No significant changes were seen in mean eGFRs between the pre- and post-intervention periods for patients in either wave. Mean (SD) eGFRs for wave I patients were 42.3 (11.9) pre-intervention and 42.1 (12.9) post-intervention (*p* = 0.71). Mean eGFRs for wave II patients were 43.9 (11.1) pre-intervention and 43.2 (11.9) post-intervention (*p* = 0.06).

#### Practice process changes

For wave I practices, priority for improving care of patients with CKD remained relatively high, with no significant change from pre- to post-intervention, and there was no significant change in subscales designed to measure organizational factors associated with practice change capacity (i.e., history of change, continuous refinement, and sustaining change), but the use of change strategies increased (Table 5). A similar pattern of findings occurred in wave II, including a significant increase in strategy count. There was, however, no statistically significant increase in practice systems in wave II practices, but their baseline scores were also higher.

**Table 5:** **Pre- and post-intervention priority, change capacity, and change process content scores**

**Instrument**	**Wave I**				**Wave II**			
	***n***	**Pre-intervention**	**Post-intervention**	***p*** value*	***n***	**Pre-intervention**	**Post-intervention**	***p*** value*
		**Mean (SD)**	**Mean (SD)**			**Mean (SD)**	**Mean (SD)**	
Priority for improving CKD care	28	6.1 (2.4)	6.4 (2.1)	0.46	39	6.0 (2.1)	6.4 (1.90)	0.27
CPCQ:								
Organizational factors	26	14.5 (11.4)	16.8 (9.0)	0.29	39	13.2 (9.8)	15.0 (7.9)	0.25
Previous history of change	28	2.4 (3.1)	3.2 (2.3)	0.25	39	2.4 (2.3)	2.5 (2.5)	0.95
Plans for organizational refinement	29	3.6 (2.6)	4.2 (1.7)	0.21	40	3.0 (2.7)	3.7 (2.0)	0.08
Ability to initiate and sustain change	26	8.3 (6.7)	9.4 (6.2)	0.43	40	8.0 (5.9)	9.1 (4.8)	0.22
Strategy count	25	3.3 (4.1)	18.6 (4.5)	<0.0001	37	5.5 (7.8)	16.7 (7.9)	<0.0001
PPC-PCMH-R:								
Health systems	28	45.8 (32.3)	50.0 (30.8)	0.27	28	41.1 (28.9)	40.5 (31.2)	0.90
Clinical information system	28	48.8 (16.0)	56.3 (16.5)	0.016	28	53.2 (16.2)	58.9 (20.2)	0.12
Decision support	28	45.8 (17.1)	63.3 (19.6)	0.0001	28	52.8 (18.4)	60.5 (24.5)	0.14
Delivery system design	25	25.0 (14.8)	34.8 (22.7)	0.013	28	32.8 (18.6)	34.4 (20.0)	0.73
Patient self-management support	28	21.8 (12.5)	32.1 (16.8)	0.005	28	26.5 (17.4)	30.8 (18.6)	0.23

*The paired *t* test was used to generate the *p* values.

*SD* standard deviation, *CPCQ* Change Process Capability Questionnaire, *PPC-PCMH* Physician Practice Connections—Patient-Centered Medical Home Research Version.

## Discussion

In their comprehensive review of innovation diffusion, Greenhalgh et al. identified a number of unanswered questions including, ‘Is there a role for a central agency, resource center, or officially sanctioned demonstration programs? and What is (or could be) the role of professional organizations and informal inter-professional networks in spreading innovation among health care organizations?’ [30] The results of this study suggest that diffusion, which is generally considered to be a passive process, can be facilitated by PBRN researchers and member practices using a combination of assistance and incentives. Whether PBRNs should assume this role or whether a broader extension system similar to cooperative extension in agriculture should be developed for this purpose remains open [31,32]. In either case, PBRNs are likely to be a source of early adopter practices and possibly clinician opinion leaders.

Our results also speak to a second question posed by the Greenhalgh review, ‘What is the nature of the networks, of different players… [and] How do these networks serve as channels for …embedding of complex service innovations?’ Most of the early adopters chose to recruit practices with similar characteristics—either members of the same health system or those that used the same EHR. Reducing the complexity of the implementation setting is likely to facilitate adoption; furthermore, health system administrators often must approve efforts such as these that require templates and order sets.

As expected, the combination of performance feedback (baseline and monthly), academic detailing, and weekly PF for 6 months resulted in improved recognition and care of patients with CKD in motivated practices. We demonstrated improvements in the use of ACEIs and ARBs, measurement of Hgb, and vitamin D testing or supplementation. Since diabetes care had received a great deal of attention already, baseline adherence to those measures was high in many practices. Likewise, rates of usage of NSAIDs were already low. It is also important to remember that 100% adherence is not an appropriate expectation, since guidelines do not apply to all patients with CKD, and we did not exclude patients with, for example, terminal illness, dementia, or contraindications to particular strategies. The interventions resulted in development of both new strategies (techniques) and new processes of care (system level changes).

Wave II practices showed improvements in care of their CKD patients similar to wave I practices with less PF assistance. This is important since PF support is relatively expensive. As in wave I, the interventions resulted in the development of new strategies, but there was no significant increase in new processes of care. However, wave II practices had less time (9 vs. 12 months) to change, and their baseline process scores were nearly as high as the post-intervention process scores for wave I practices, leaving less room for improvement. This could have, in part, been due to cross-contamination within health systems that chose to implement system wide changes prior to wave II.

We are optimistic that many of the new processes implemented during this project will be sustained since they were typically accompanied by the creation of templates, order sets, and other modifications to the EHRs. The automatic calculation of eGFR by the labs used by participating practices prior to or as a result of the project, and the increased awareness of the importance of eGFR created by the project are also likely to persist. However, at this point, we have no data upon which to assess sustainability. Despite the availability of EHR options like templates and order sets, the one-page summary of guideline recommendations organized by CKD stage and substage proved to be very helpful to practices as they developed strategies for improvement (see Additional file 1). Many practices reported putting them in exam rooms and referring to them routinely. The one-page summary was also provided to several practices not involved in the project upon request after having heard about it from colleagues. This we found interesting, since, in theory, all of this could be programmed into an EHR.

We found evidence that putting CKD on the problem list is a critical first step in providing evidence-based care for patients with CKD. Guideline producers should consider developing both practice-friendly summaries and suggestions regarding implementation sequencing. This would require collaboration with end-users (practices) and implementation researchers. PBRNs could be helpful in this role.

This study has several limitations. It was designed to determine whether experienced practices could facilitate improvements in other practices. A separate control group was not included. Therefore, a secular trend toward better care of CKD patients could have contributed to observed performance improvements. However, practices engaged in the project uniformly indicated that they had done little to improve their care of patients with CKD prior to the project, and we know of no attempts by others in the involved states to address this problem during the project. Likewise, we are unaware of changes in medication cost or availability that might have resulted in increased prescribing of ACEIs or ARBs, though that is certainly a possibility.

Due to delays in recruitment and enrollment of wave II practices and the prohibition on no-cost extensions imposed by the American Recovery and Reinvestment Act, the length of the wave II post-intervention period was shortened from 12 months (as in wave I) to 9 months. As a result, improvement in measures of guideline adherence requiring annual testing (e.g., measurement of lipids, anemia tests, and microalbumin) may be underestimated. Wave II practices also had less time to implement system level changes which could have reduced the impact of the intervention.

We anticipated abstracting data on 30 patients from each participating practice. Unfortunately, practices varied widely in their ability to identify patients with two eGFRs <60 at least 3 months apart. Therefore, the number of patients from practices eligible for these analyses ranged from 1 to 27, making it impossible to adjust for variation among practices. Although the smaller than expected sample reduced the statistical power of our analyses, we were nevertheless able to demonstrate significant improvements in guideline implementation by both wave I and wave II practices.

## Conclusions

After receiving implementation assistance, early adopter practices were able to help to recruit and support additional practices with implementation of the National Kidney Foundation Kidney Disease Outcomes Quality Initiative CKD guidelines through monthly LLCs, performance feedback (baseline and monthly), academic detailing, and monthly practice facilitation over a 6-month period. This may be an important way to disseminate, implement, and diffuse other evidence-based innovations.

## Additional file

Additional file 1:
**Summary of CKD guideline recommendations for primary care (updated).**

## Abbreviations

A1c: Hemoglobin A1c
ACEIs: Angiotensin converting enzyme inhibitors
ARBs: Angiotensin receptor blockers
CI: Confidence intervals
CKD: Chronic kidney disease
CME: Continuing medical education
CPCQ: Change process capability questionnaire
DBP: Diastolic blood pressure
eGFR: Estimated glomerular filtration rates
EHR: Electronic health record
GEE: Generalized estimating equations
Hgb: Hemoglobin
LDL: Low-density lipoprotein
LLCs: Local learning collaboratives
microalbumin: Urine albumin/creatinine ratio
MOC: Maintenance of certification
NSAIDS: Non-steroidal anti-inflammatory medications
OKPRN: Oklahoma physicians resource/research network
OR: Odds ratio
PBRN: Practice-based research networks
PFs: Practice facilitators
PPC-PCMH-R: Physician practice connections – patient-centered medical home research version
QI: Quality improvement
SBP: Systolic blood pressure
