# Supplementary material for: Leveraging practice-based research networks to accelerate implementation and diffusion of chronic kidney disease guidelines in primary care practices: a prospective cohort study
Source: Implement Sci. 2014 Nov 23;9:169. doi: 10.1186/s13012-014-0169-x (PMC4245828; doi:10.1186/s13012-014-0169-x)
Supplement: Additional file 1: — Summary of CKD guideline recommendations for primary care (updated). [file 13012_2014_169_MOESM1_ESM.doc]

Summary of CKD guideline recommendations for primary care (updated)

| eGFR < 60 | eGFR < 45 | eGFR < 30 |
| --- | --- | --- |
| Documentation: CKD on problem list 585.3 | Documentation: CKD on problem list 585.3 | Documentation: CKD on problem list 585.4 |
| Lab tests: annual • Urinalysis • Urine microalbumin • A1c (6 months) • Lipid panel | Lab tests: annual • Urinalysis • Urine microalbumin • A1c (6 months) • Lipid panel • Calcium, phosphorus • PTH (intact molecule)* • 25-hydroxy vitamin D • CBC • Iron/TIBC (if Hb <12.0) | Lab tests: annual • Urinalysis • Urine microalbumin • A1c (6 months) • Lipid panel • Calcium, phosphorus • PTH (intact molecule)* • 25-hydroxy vitamin D • CBC • Iron/TIBC (if Hb < 12.0) |
| Current meds: • NSAIDs (D/C) • Bisphosphonates • Allopurinol (reduce dose) • IV contrast (caution) • Metformin (Caution) | Current meds: • NSAIDs (D/C) • Bisphosphonates • Allopurinol (reduce dose) • IV contrast (caution) • Metformin (caution) | Current Meds: • NSAIDs (D/C) • Bisphosphonates (D/C) • Allopurinol (reduce dose) • IV contrast (caution) • Metformin (caution) |
| New meds: • ACEI or ARB • ASA 81 mg (consider) • Vit. D3, 2,000 IU QD (consider) | New meds: • ACEI or ARB • ASA 81 mg (consider) • Vit. D3, 2,000 IU QD (consider) | New meds: • ACEI or ARB • ASA 81 mg (consider) • Vit. D3, 2,000 IU QD (consider) |
| Risk factor management: • BP <140/80 • A1c <7 (if diabetic) LDL < 100^7^ | Risk factor management: • BP <140/80 • A1c <7 (if diabetic) • LDL <100 • Calcium >8.5 • PO4 < 4.6 • PTH <100 • 25-OH Vit D >30 • Hemoglobin >10 and <12 | Risk factor management: • BP <140/80 • A1c <7 (if diabetic) • LDL <100 • Calcium >8.5 • PO4 < 4.6 • PTH <100 • 25-OH Vit D >30 • Hemoglobin >10 and <12 |
|  | Vein preservation: • Blood draws from one arm • No PICC Lines or place in neck | Vein preservation: • Blood draws from one arm • No PICC Lines or place in neck |
| Referral: N/A | Referral: N/A | Referral: Nephrologist |
| Other: • Flu vaccine (yearly) • Pneumococcal vaccine • Smoking cessation counseling • Patient education form | Other: • Flu vaccine • Pneumococcal vaccine • Smoking cessation counseling • Patient education form | Other: • Flu vaccine • Pneumococcal vaccine • Hepatitis B vaccination • Smoking cessation counseling • Patient education form |

* Correction of vitamin D may correct PTH.
